# Enhanced anti-inflammatory and ulcerogenicity of Ibuprofen microsphere formulations using Irvingia wombolu fat (IRW) and moringa oil (MO) as co-lipids

**DOI:** 10.1186/s12906-023-04036-2

**Published:** 2023-07-19

**Authors:** Thaddeus H. Gugu, Geraldine C. Agu, Emmanuel M. Uronnachi, Salome A. Chime

**Affiliations:** 1 Drug Delivery Unit, Department of Pharmaceutical Microbiology and Biotechnology, University of Nigeria, Nsukka, Enugu State Nigeria; 2 Department of Pharmaceutical Technology and Industrial Pharmacy, University of Nigeria, Nsukka, Enugu State, Nigeria; 3 Department of Pharmaceutics and Pharmaceutical Technology, Nnamdi Azikiwe University, Awka, Anambra State Nigeria

**Keywords:** Ibuprofen, Lipid-microspheres, Anti-inflammation, Ulcerogenicity, Bioavailability

## Abstract

Ibuprofen is a member of the propionic acid class of nonsteroidal anti-inflammatory drugs (NSAIDs) with anti-inflammatory, analgesic, and antipyretic activities used to relieve a variety of pains. The objective of this study was to formulate, characterize and evaluate the in vitro and in vivo properties of ibuprofen formulated as solid lipid microspheres (SLMs) for enhanced delivery. The mixtures of *Irvingia wombolu* fat (IRW) and moringa oil (MO) each with Phospholipon® 90G (PL90G) at the ratio of 2:1 w/w were prepared by fusion, characterized and used to prepare SLMs. The SLMS were thereafter evaluated using the following parameters: particle size and morphology, stability, and encapsulation efficiency EE (%). In vitro release was carried out in phosphate buffer (pH 7.4). The ibuprofen based SLMs were also evaluated for anti-inflammatory and anti-ulcer effects using animal models. The pH showed significant increase after two months of formulation with a maximum value of 6.4 while the EE obtained were 95.6, 89.4 and 61.6% for SLMs formulated with lipid matrix of Phospholipon® 90G (1% and 2%), and MO (1%) respectively. The in vitro release showed maximum release of 87.8 and 98.97% of the two different lipid-based formulations while anti-inflammatory effect was up to 89.90% after 5 h of inducing inflammation. The SLMs did not show any lesion thus conferring gastroprotection on the formulations. The SLMs exhibited good anti-inflammatory property with gastroprotective action.

## Introduction

Ibuprofen is a propionic acid class of nonsteroidal anti-inflammatory drugs simply called NSAIDs with anti-inflammatory, analgesic, and antipyretic activities. It is used in the management of a wide range of different pains such as muscular pain and rheumatic pain [1]. Also, ibuprofen is one the leading NSAIDs used in certain illnesses like headaches, cold, backache, dysmenorrheoa, migraine neuralgia, arthritis, fever and other flu symptoms [2]. Recent research has shown that NSAIDs such as ibuprofen and others have a potential effect in the treatment of Alzheimer's disease [3]. The wound healing properties of ibuprofen and its side effects, as well as its efficacy have been extensively investigated [4, 5]. It undergoes rapid bio-transformation with a serum half-life of about 1.5 to 2 h thus leading to a short duration of action. Like other NSAIDs, it causes gastric irritation which in most cases, led to gastrointestinal damage [6]. In order to overcome this limitation, ibuprofen-loaded lipid microspheres were considered to improve solubility and mucosa absorption for maximum biodistribution and bioavailability into the systemic circulation while offering reduced inflammation and gastric irritation. Lipid microspheres, also known as lipospheres, are regarded as drug carrier systems consisting of water and lipids dispersions to form a solid-based system with a particle size range of 0.01 to 100 μm. They possess a hydrophobic lipid core stabilized by layers of phospholipid molecules around their surface. The active drugs are usually dispersed in the lipid matrix which form the internal core to enhance solubility [7], and subsequently increase plasma concentration thus improving bioavailability. Lipid-based systems are widely used in drug delivery. They enhance the bioavailability and therapeutic index of many compounds, especially poorly water-soluble drugs. They are a commercially viable approach in formulating pharmaceutical dosage forms for different routes of administration such as parenteral, oral or topical delivery [8]. Solid lipid lipospheres have earlier been investigated as a system for oral drug delivery of insulin using a methacrylic acid-based microparticulate hydrogel formulation [9]. Similarly, previous researchers have reported the delivery of ibuprofen using synthetic lipid materials [10]. *Irvingia wombolu* (Irvingiaceae) is a tropical African tree that grows in local settings across West and Central Africa and is used extensively as fruit and a thickener in food preparations [11]. *Irvingia wombolu* fat is a natural fat extracted from the kernel seeds of *Irvingia wombolu* which has been used in cosmetics and also, as a potential lipid carrier in drug delivery [12]. Moringa oil on the other hand, is also a natural oil extracted from the *Moringa oleifera* Linn. (Moringaceae) plant spread across the tropical and subtropical regions of the globe, especially the India subcontinent where it is mostly used in diet for its medicinal value [13]. The moringa seed is reported to contain up to 47% oil which is rich in oleic acids and other monounsaturated fatty acids [14]. The substitution of synthetic lipids with natural product materials such as *Irvingia wombolu* fat and moringa oil as co-lipids is an alternative worth exploring to reduce the toxicities or incompatibilities that may be associated with synthetic materials.

Solid lipid particles combine several advantages and avoid the disadvantages of other colloidal carriers. They provide a condition for drug targeting and controlled drug release and also, protect the loaded active compounds against enzymatic or chemical degradation and gastric irritation [15]. The solid matrix is made up of physiologically compatible lipids which allow hydrophilic or hydrophobic drugs to be incorporated and form a vesicular core [16, 17]. These lipid-based particulate drug carriers exist as nano-carriers, microparticulate and colloidal carriers [18]. Non-steroidal anti-inflammatory drugs (NSAIDs) today, have been considered for delivery in nano- or micro-sized particles for solubility enhancement and gastroprotection using lipid matrixes to reduce /prevent a rise in gastric intestinal tract (GIT) disorders such as ulcerative lesions, colitis and colon polyps which may be aggravated by the direct contact of these agents with the lining tissues of the GIT [19]. This research seeks to optimize the delivery of ibuprofen to enhance solubility, biodistribution and bioavailability with natural based lipid carrier systems for patient compliance and limited GIT mucosal cell injury. Ibuprofen was formulated into lyophilized and reconstitutable parenteral powder as ibuprofen solid lipid microspheres (Ib-SLMs). The consideration of dry *micro* and *nano* crystalline powder formulation of these poorly water-soluble drugs, has over the years proven to be more efficient in systemic drug delivery and bioavailability for a quicker onset of action [20–22].

## Materials and methodology

### Materials

The following materials were used for the study. Ibuprofen (Juhel Pharmaceutical Ltd, Nigeria), mixtures of *Irvingia wombolu* fat (IRW) and moringa oil (MO) gotten from Natural product unit, Department of Pharmaceutical and Medicinal Chemistry, University of Nigeria Nsukka. All other chemicals and reagents used were of analytical grade.

#### Formulation composition

The percentage composition of the formulations is expressed in Table 1.

**Table 1:** Percentage ratio of raw materials in formulations

Batch code	Lipid* matrix (%)	Ibuprofen (%)	Soluplus® (%)	Sorbitol (%)	Sorbic acid (%)	Distilled water qs (%)
A1	5	1	2	4	0.05	100
A2	5	1.5	2	4	0.05	100
A3	5	2	2	4	0.05	100
A4	5	0	2	4	0.05	100
B1	5	1	1	4	0.05	100
B2	5	1.5	1	4	0.05	100
B3	5	2	1	4	0.05	100
B4	5	0	1	4	0.05	100

Key: ^*^Contains lipid matrix combinations in the ratio of 2:1: A1-A3 contains *Irvingia wombolu* fat (IRW) and Moringa oil (MO) respectively; B1-B3 contain *Irvingia wombolu* fat and Phospholipon® 90G (PL90G) respectively

### Methodology

#### Formulation of lipid matrix

Mixtures of *Irvingia wombolu* fat (IRW) with moringa oil (MO) (Natural product unit, department of Pharmaceutical and medicinal chemistry, University of Nigeria Nsukka with Phospholipon® 90G (Phospholipid Gmbh, Koln, Germany) (2:1 w/w) respectively, were prepared by fusion. The varying lipids as presented in the table above were weighed out using an analytical weighing balance (Adventurer, Ohaus, China), melted together and stirred at a temperature of 70 °C using a magnetic stirrer (SR1 UM 52188, Remi Equipments, India), until a completely homogenous and transparent white mixture was obtained. The homogenous mixture was stirred at room temperature until solidification to obtain the solid lipid matrix [23].

#### Preparation and lyophilization of Ib-SLMs

The ibuprofen-loaded lipid microspheres were prepared using the melt homogenization technique [17], following the formula in Table 1 above. In each case, 5 g of the lipid matrix (LM) was melted using Ultra-Turrax homogenizer (T25 Basic Digital, Ika, Staufen, Germany) at 70 ºC and an appropriate amount of Ibuprofen was incorporated into the melted lipid. Also, sorbitol (Wharfedale Laboratories, Otley, West Yorkshire) was dissolved in hot distilled water at the same temperature with the lipid melt together with Soluplus® (BASF corporation, USA) to obtain an aqueous phase. The hot aqueous phase was poured into the lipid melt and immediately subjected to high shear homogenization with Ultra-Turrax at 5000 rpm for 10 min. An o/w emulsion was obtained by phase inversion. All batches of the formulation were lyophilized using a freeze-dryer (Amsco/Finn–Aqua_Lyovac GTZ, Hu¨rth, Germany) to obtain the resultant solid particles for further characterization and analysis.

##### Percentage yield

The percentage yield (% w/w) of the resultant solid lipid-microspheres prepared was determined to obtain the extent of the miscibility and homogenization of the mixture using equation 1.

1 $$\mathrm{Percentage}\;\mathrm{recovery}\;(\%)=\frac{\mathrm W1}{\mathrm W2+\mathrm W3}\times100$$

Where *w*_*1*_ is weight of SLMs formulated (g), *w*_*2*_ is the weight of drug added (g) and *w*_*3*_ is the weight of raw materials {lipid, sorbitol, sorbic acid and soluplus® (g)}.

##### Time-dependent pH stability studies

The pH of both the lyophilized and un-lyophilized formulations was studied in a time-dependent manner: 24 h, 1 week, 2 weeks, 3 weeks, 1 month, and 2 months using a pH meter (pH ep® Hanna instrument, Padova, Italy) to check the stability of the formulations [24]. The pH meter was first calibrated with standard pH solutions of pH 4, 7 and 10. Afterwards, the pH of the dispersions was determined by inserting the electrode of the instrument into the dispersion of the formulation.

##### Particle size and morphology analysis

The bottles containing the different batches of the formulations were shaken vigorously and were syringed out using a 5 ml syringe. Two drops of the different formulations were dispensed on a clean slide, covered with a cover slip and examined under a binocular microscope attached with a motic image analyzer at a magnification of X 400. For the lyophilized lipid-microspheres, about 200 mg of the lipid-microspheres from each batch was dispersed in a small amount of water and placed on a slide, covered with a cover slip and also examined under a binocular microscope at a magnification of X 400.

##### Drug content determination

Quantities of lipid microspheres equivalent to 0.1 g of ibuprofen were weighed out and placed in a 100 ml volumetric flask. The flask was made to the volume with phosphate buffer and heated at 70 ºC with intermittent shaking until the lipid microspheres completely melted. The dispersion was cooled at room temperature and filtered through filter paper. Ibuprofen content of appropriate dilutions were analyzed spectrophotometrically using UV spectrophotometer (Spectrum laboratory, The Netherlands) at a predetermined wavelength at 287 nm. This was repeated three times for all the batches. The drug concentrations were calculated with reference to a Beer’s plot.

##### Entrapment efficiency (EE %) of Ib-SLMs

The quantities of the drug theoretically contained in the lipid-microspheres were compared with the quantity actually obtained from the drug content studies. This was calculated using the Eq. (2).

2 $$\mathrm{Entrapment}\;\mathrm{efficiency}\;(\%)=\frac{\mathrm{Amount \;of \;drug}\;\left(\mathrm{Ib}\right)\;\mathrm{remaining \;in \;SLMs}}{\mathrm{Total \;amount \;of \;drug}\;\left(\mathrm{Ib}\right)\;\mathrm{added \;into \;the \;SLMs}}\mathrm X \;100$$

##### Loading capacity (LC)

Loading capacity (LC) expresses the ratio between the entrapped active pharmaceutical ingredient (API) and the total weight of the lipids [25]. LC was determined using the relationship.

3 $$\mathrm{Loading}\;\mathrm{capacity}\;(\%)=\frac{\mathrm{Amount}\;\mathrm{of}\;\mathrm{entrapped\;\mathrm{drug\;(Ib)}\;\mathrm{in}\;SLMs}}{\mathrm{Total}\;\mathrm{weight}\;\mathrm{of}\;\mathrm{SLMs}}\mathrm X \;100$$

##### In vitro release studies

Beer’s plot was obtained for ibuprofen in phosphate buffer (pH 7.4) at a concentration range 0.1–1.0 mg% at a pre-determined wavelength of 287 nm. The in vitro release kinetics was determine following an established protocol in dissolution medium consisting of 250 ml offreshly prepared medium, phosphate buffer (pH 7.4) maintained at 37 ± 1 ºC [26]. The polycarbonate dialysis membrane (MWCO 6000–8000, Spectrum Labs, Breda, the Netherlands) selected was pre-treated by soaking in the dissolution medium for 24 h prior to use. A quantity of the lipid particles equivalent to 0.1 g of ibuprofen was weighed from each batch and placed in a polycarbonate dialysis membrane containing 2 ml of the dissolution medium, securely tied with a thermo-resistant thread and placed in the appropriate chamber on the magnetic stirrer (SR1 UM 52188, Remi Equipment Mumbai, India) as release apparatus. The paddle was rotated at 100 rpm, and at pre-determined timed intervals, 5 mL portions of the dissolution medium was withdrawn, appropriately diluted and analyzed for drug content in a spectrophotometer. The volume of the dissolution medium was kept constant by replacing it with 5 mL of fresh medium after each withdrawal to maintain sink condition. The amount of drug released at each time interval was determined with reference to Beer’s plot.

##### Anti-inflammatory studies

The anti–inflammatory activity of the ibuprofen loaded lipid microspheres was carried out using the rat paw oedema test [27]. All the protocols were approved and carried out in accordance with guidelines and regulations of the Animal Ethics Committee, Faculty of Pharmaceutical Sciences, University of Nigeria, Nsukka with ethical approval number of UNN/FPS/20/0025a. The experimental animals were procured from the animal facility of the Department of Veterinary Medicine, University of Nigeria, Nsukka and allowed access to water. The phlegmatic agent employed in the study was fresh undiluted egg albumin. Adult Wister rats of either sex (96—150 g) were divided into four experimental groups of three rats per group. The animals were fasted and deprived of water for 12 h before the experiment. The deprivation of water was to ensure uniform hydration and to minimize variability in oedematous response [28]. The ibuprofen-loaded lipid-microspheres of the different batches (A1 and B1) equivalent to 6 mg/kg body weight was administered orally to the rats. The reference group (positive control) received 6 mg/kg of the pure sample of ibuprofen, while the control group (negative control) received normal saline. Thirty minutes’ post-treatment, oedema was induced by the injection of 0.1 ml fresh undiluted egg–albumin into the sub plantar region of the right hind paw of the rats. The volumes of distilled water displaced by treated right hind paw of the rats were measured using a plethysmometer before injection, and at 0.5, 1, 2, 3, 4, 5 and 6 h after egg albumin injection. The average oedema at every interval was assessed in terms of difference in volume displacement (Vt-Vo), while percent inhibition of oedema was calculated using the equation below [29].

4 $$\mathrm{Percentage}\;\mathrm{oedema }\;\mathrm{inhibition\;(I)}=\frac{\left(\mathrm{dt}\right)}{\left(\mathrm{dc}\right)}\times100$$

Where *dt* is the difference in paw volume of the animals treated with drug-treated group and *dc* is the difference in paw volume in control group, while *I* stand for inflammatory inhibition.

##### Ulcerogenicity studies

The ulcerogenicity of ibuprofen-loaded lipid-microspheres was determined using the gastric mucosa irritation animal model method as described in earlier studies [30]. The studies were carried out on healthy Wistar rats (96 – 150 g). The animals were divided into four experimental groups of three animals per group. The control group received normal saline while the reference group received 6 mg/kg of pure sample of ibuprofen orally. The animals were fasted for 8 h prior to administering a single dose of either the control or the test compounds, given free access to food and water, and sacrificed 17 h later by ether anesthesia. The gastric mucosa of the rats was examined under a microscope using a X 4 binocular magnifier. The lesions were counted and divided into large (greater than 2 mm in diameter), small (1–2 mm) and puntiform (less than 1 mm). For each stomach, the severity of mucosal damage was assessed according to the following scoring system [31]; 0 – no lesions or one puntiform lesion; 1 – two to five puntiform lesions; 2 – one to five small ulcers; 3 – more than five small ulcers or one large ulcer; and 4 – more than one large ulcer. All the experimental methods were reported in accordance with the ARRIVE guidelines.

## Results and discussion

### Percentage yield

**Fig. 1:**
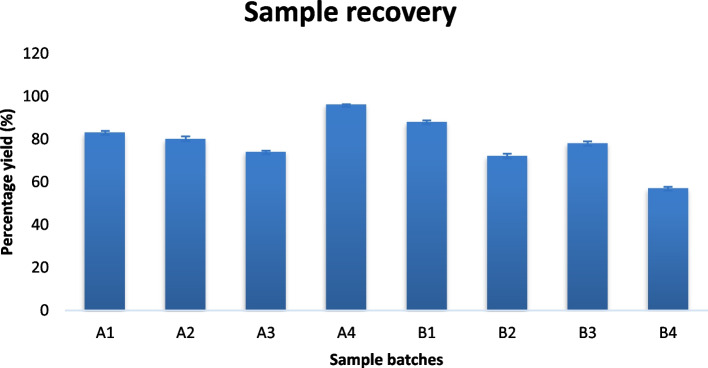
Percentage recovery of lipid microspheres; A1-A3 contains LM *Irvingia wombolu* fat and moringa oil, B1-B3 contains LM *Irvingia wombolu* fat and Phospholipon® 90G and different concentrations of ibuprofen while batches A4 and B4 contain no API, (*n* = 3)

The result of percentage recovery of the lipid microspheres as seen in the Fig. 1 showed a good yield of the formulations for both loaded and unloaded lipid-microspheres (batch A1-A4 and B1-B4). There was a high percentage recovery ranging from 57 – 96%.

### Time-dependent pH stability studies

The result of pH stability of the un-lyophilized lipid microspheres as shown in Table 2, showed that after 2 months, all the batches formulated with the lipid matrix of *Irvingia wombolu* fat and moringa oil (batches A1-A4) had a pH in the range of 5.0 ± 0.04 to 7.2 ± 0.02 and the batches formulated with *Irvingia wombolu* fat and Phospholipon® 90G (batches B1-B4) had a pH of 4.8 ± 0.01 to 6.0 ± 0.05. Changes in pH of the unlyophilized formulations may be due to degradation of excipients. Lyophilizing the formulation and the possible inclusion of a buffer may be used to maintain the pH.

**Table 2:** pH result before lyophilization

**Batch code**s	**1 day**	**1 week**	**2 weeks**	**3 weeks**	**1 month**	**2 months**
A1	5.3 ± 1.2	5.6 ± 1.3	5.6 ± 1.3	6.2 ± 0.03	6.2 ± 0.03	6.1 ± 0.03
A2	5.2 ± 2.0	5.5 ± 1.2	5.4 ± 1.0	5.8 ± 0.4	6.0 ± 0.05	6.0 ± 0.05
A3	5.0 ± 0.8	5.4 ± 1.0	5.3 ± 1.2	6.3 ± 0.04	6.4 ± 0.03	6.0 ± 0.05
A4	5.4 ± 1.3	5.7 ± 1.2	5.4 ± 1.5	7.2 ± 0.03	7.0 ± 0.01	6.3. ± 0.1.2
B1	5.1 ± 1.0	5.1 ± 1.0	5.0 ± 1.2	5.4 ± 1.4	5.5. ± 1.2	5.6 ± 1.3
B2	5.0 ± 0.4	4.9 ± 0.6	4.8 ± 1.3	5.2 ± 1.3	5.4 ± 1.0	5.0 ± 1.5
B3	5.0 ± 0.4	5.0 ± 0.4	4.9 ± 2.2	5.6 ± 1.2	5.7 ± 1.0	5.5 ± 1.3
B4	5.3 ± 1.2	5.2 ± 1.0	5.1 ± 1.0	5.7 ± 1.2	5.8 ± 0.4	6.0 ± 0.05

Key: A1-A3 contains LM *Irvingia wombolu* fat and moringa oil, B1-B3 contains LM *Irvingia wombolu* fat and Phospholipon® 90G and different concentrations of ibuprofen while batches A4 and B4 contain no API, (*n* = 3). Results presented as mean ± SD

### The pH profile of lyophilized lipid microspheres dispersion

The result of the pH stability after lyophilizing the lipid-microspheres dispersion as shown in Table 3, show the pH to be in the range of 6.0 ± 0.05 to 6.4 ± 0.03. This is within the pH range of the pure ibuprofen which is 6.2 ± 0.03. A prior stable drug may be affected by degradation of excipients with storage through generation of an unfavourable pH (increase or decrease) or reactive species for the drug. However, the slight increase in the pH values in most lipid-microspheres formulation was not attributed to drug degradation since there was an increase in the pH of the unloaded lipid-microspheres.

**Table 3:** The pH result after lyophilized lipid microspheres dispersion

Batch code	pH value
A1	6.4 ± 0.21
A2	6.4 ± 0.13
A3	6.2 ± 0.21
A4	6.4 ± 0.22
B1	6.0 ± 0.16
B2	6.2 ± 0.31
B3	6.0 ± 0.15
B4	6.2 ± 0.27
C	6.2 ± 0.13

Key: A1-A3 contains LM *Irvingia wombolu* fat and moringa oil, B1-B3 contains LM *Irvingia wombolu* fat and Phospholipon® 90G and different concentrations of ibuprofen while batches A4 and B4 contain no API, Batch C is the pure sample of ibuprofen, (*n* = 3). Results presented as mean ± SD

### Differential scanning calorimetry (DSC)

**Fig. 2:**
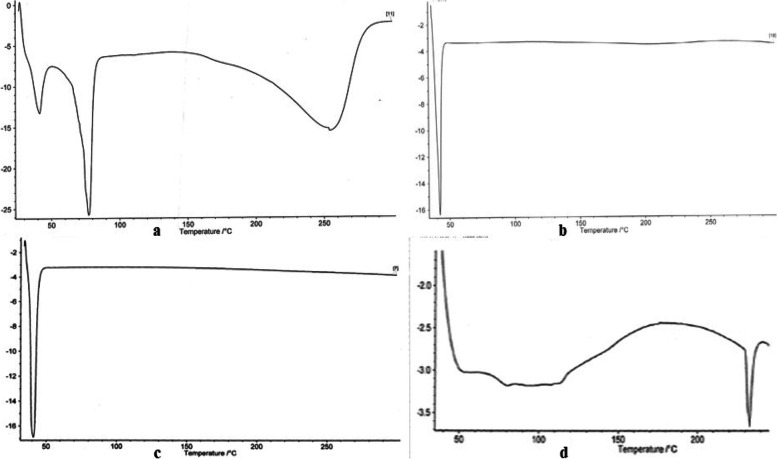
The DCS thermograms: **a.** Ibuprofen drug, **b.** *Irvingia wombolu* fat, **c.** Moringa oil, **d.** Phospholipon® 90G

**Fig. 3:**
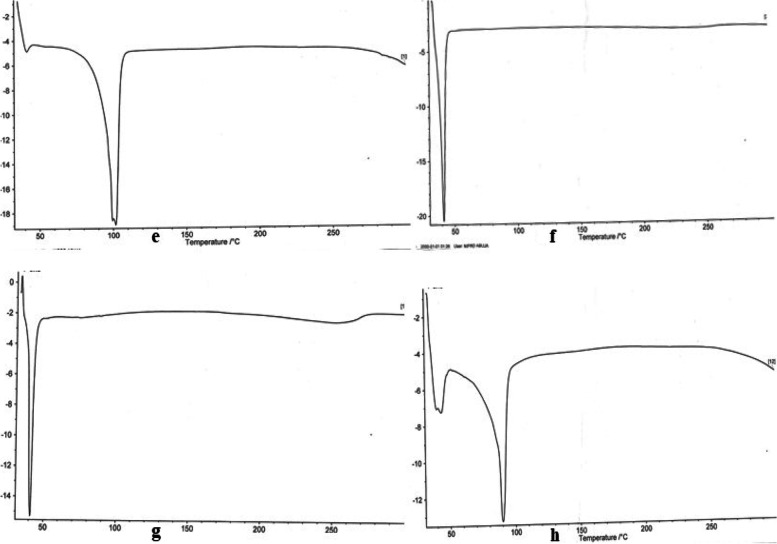
The DCS thermograms: **e** Lipid matrix *irvingia wombolu* fat and phospholipon® 90, **f** Lipid matrix *Irvingia wombolu* fat and moringa oil, **g** 1% ibuprofen loaded liposphere containing lipid matrix *Irvingia wombolu* fat and moringa oil, **h** 1% ibuprofen-loaded liposphere containing lipid matrix *Irvingia wombolu* fat and phospholipon® 90G

The DSC thermograms in Figs. 2 and 3 show a sharp endothermic point at 77.1ºC which is in agreement with the melting point of ibuprofen with the range of 75 to 78ºC as contained in British Pharmacopeia (BP), therefore indicating presence of pure crystalline ibuprofen. The lipid matrix employed in the formulation of lipid-microspheres, when scanned with the DSC traced various peaks due to the lipid content of the matrices which contains fatty acids and steroids of different endothermic peak points and functions as a structural components of cell membranes [32, 33]. The lipid-microspheres containing lipid matrix in Fig. 2b, c and d of *Irvingia wombolu* fat and moringa oil gave a lower enthalpy at 41.4 ºC.

### Particle size

Particle size distributions were extrapolated and calculated in Table 4. The lipid-microspheres formulated with the highest concentration of ibuprofen (2%) in batch A3 and B3 showed highest mean particle size in each batch with 23.97 ± 0.20 and 19.74 ± 0.21 µm respectively.

**Table 4:** Particle size analysis of lipid microspheres

Batch code	Particle size (µm)	
	**Before lyophilization (µm ± SD)**	**After lyophilization (µm ± SD)**
A1(a)	22.56 ± 0.39	21.15 ± 0.99
A2(b)	18.33 ± 0.67	9.85 ± 0.99
A3(c)	23.97 ± 0.20	25.38 ± 0.39
A4(d)	19.74 ± 0.19	18.33 ± 0.10
B1(e)	16.92 ± 0.19	25.38 ± 0.40
B2(f)	16.92 ± 0.19	36.66 ± 0.58
B3(g)	19.74 ± 0.21	18.33 ± 0.21
B4(h)	16.92 ± 0.19	18.33 ± 0.10

Key: A1-A3 contains Lipid matrix, *Irvingia wombolu* fat and moringa oil, B1-B3 contain LM *Irvingia wombolu* fat and Phospholipon® 90G and different concentrations of ibuprofen while batches A4 and B4 contain no API, (*n* = 3). Results presented as mean ± SD

**Fig. 4:**
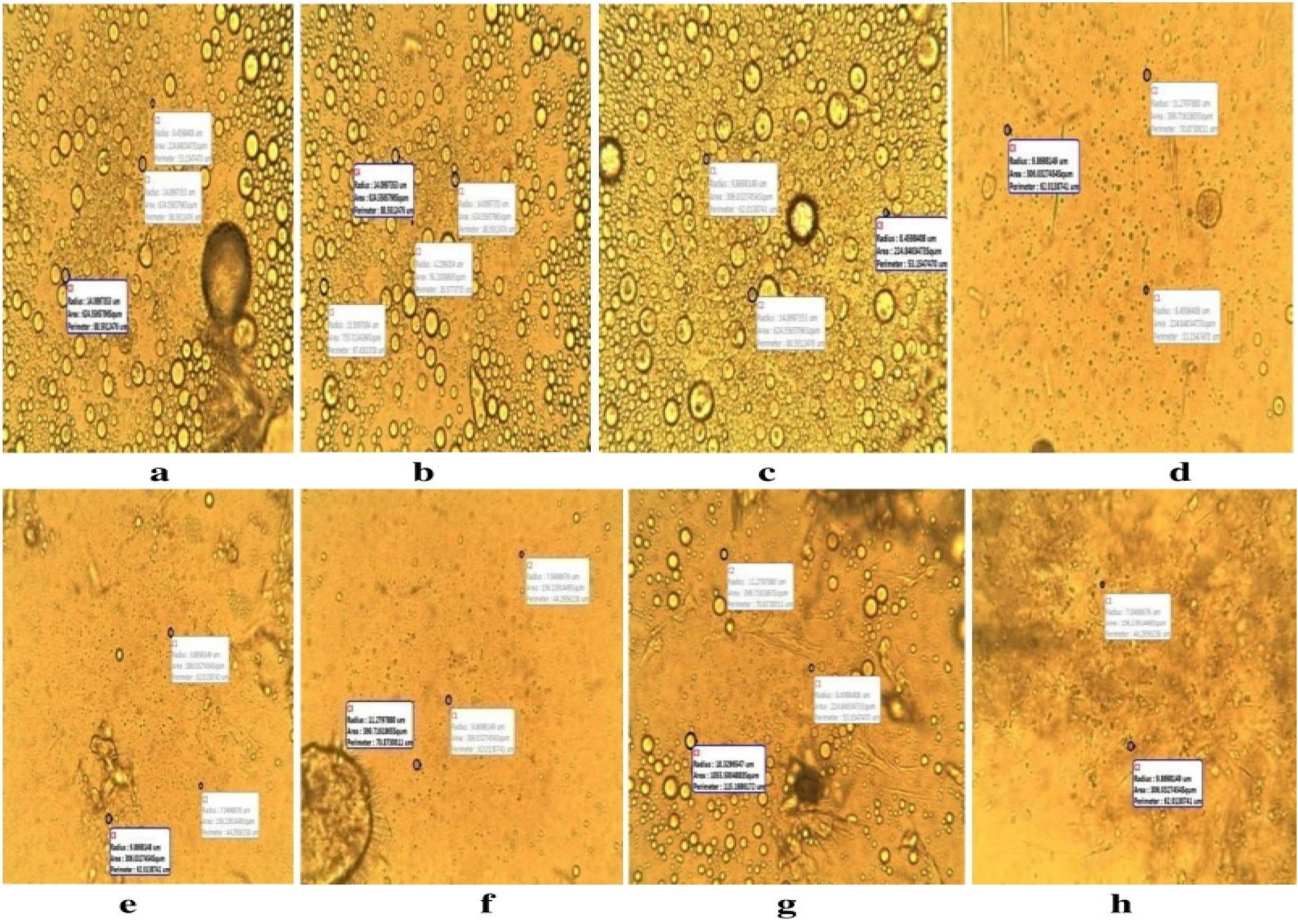
Photomicrographs of liposphere before lyophilization: (**a**) 1% ibuprofen lipid microspheres with IRW and MO, (**b**) 1.5% ibuprofen lipid-microspheres with IRW and MO, (**c**) 2% ibuprofen with IRW and MO, (**d**) bland lipid-microspheres with IRW and MO, (**e**) 1% ibuprofen lipid-microspheres with IRW and PL90G, (**f**) 1.5% ibuprofen lipid-microspheres with IRW and PL90G, (**g**) 2% ibuprofen lipid-microspheres with IRW and PL90G, (**h**) bland ibuprofen lipid-microspheres with IRW and PL90G, IRW = *Irvingia wombolu* fat, MO = moringa oil, PL90g = Phospholipon® 90G

Figure 4 showed the photomicrograph of the unlyophilized lipid microspheres with different sizes. The various sizes and morphologies could possibly be as a result of shear force applied during formulation as earlier reported [34], this subsequently enhanced the breakdown of larger particles into smaller sizes. Furthermore, the nature of lipids used in formulations could have affected sizes obtained [35]. Batch A formulated with the lipid matrix of *Irvingia wombolu* fat and moringa oil, showed a higher mean particle size ranging from 18.33 ± 0.67—23.97 ± 0.20 µm than batch B which was formulated with *Irvingia wombolu* fat and Phospholipon® 90G with particle size ranging from 16.92 ± 0.19—19.74 ± 0.21 µm.

### Entrapment efficiency (EE%) and loading capacity (LC)

The result in Table 5 represents the EE % and the LC of various batches of ibuprofen-loaded lipid-microspheres. The EE% showed that the lipid-microspheres loaded with 1% ibuprofen (batches A1 and B1) had highest EE% of 89 and 95% respectively. Also, the loading capacity obtained for ibuprofen-loaded lipid-microspheres ranged from 8.94 – 12.88 g/100 g lipid for A batches, and 9.56 – 12.32 g/100 g lipid for B batches. Batches A3 and B3 with 2% ibuprofen gave the highest LC for each batch, 12.88 and 12.32 respectively.

**Table 5:** The Entrapment efficiency and loading capacity

Batch code	EE (%)	LC
A1	89	8 ± 0.94
A2	81	11 ± 0.93
A3	32	12 ± 0.88
B1	95	9 ± 0.56
B2	91	10 ± 0.52
B3	61	12 ± 0.32

Key: batch A1, A2 and A3 contains LM *Irvingia wombolu* fat and moringa oil loaded with 1, 1.5 and 2% of ibuprofen respectively, batch B1, B2 and B3 contains LM *Irvingia wombolu* fat and Phospholipon® 90G loaded with 1, 1.5 and 2% of ibuprofen respectively, (n = 3 ± SD)

### In vitro release profile of ibuprofen lipid-microspheres in phosphate buffer

**Fig. 5:**
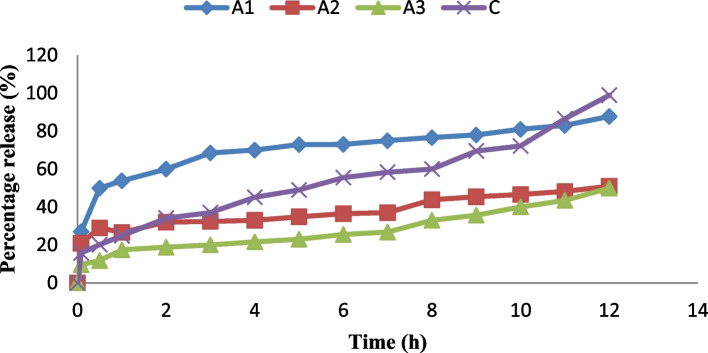
The in vitro release profile of ibuprofen of the liposphere formulated. A1-A3 contains LM *Irvingia wombolu* fat and moringa oil with different concentrations of ibuprofen and C is pure sample of ibuprofen, (*n* = 3)

**Fig. 6:**
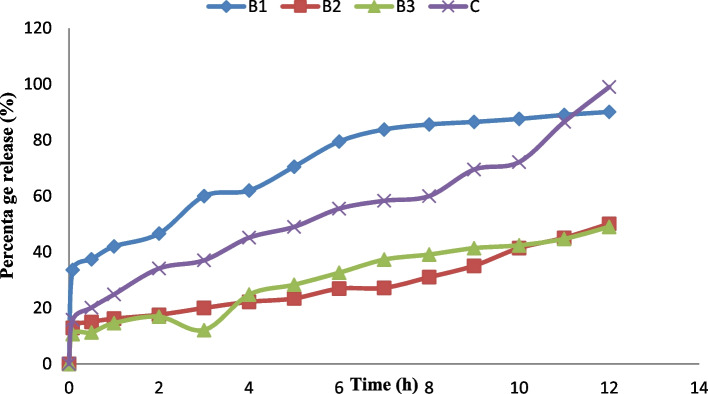
The in vitro release profile of ibuprofen of the lipid-microspheres formulated. B1-B3 contain LM *irvingia wombolu* fat and phospholipon® 90G with different concentrations of ibuprofen and C is pure sample of ibuprofen, (*n* = 3)

The result of in vitro release showed in Figs. 5 and 6 indicated that the release kinetics of ibuprofen in phosphate buffer exhibited a very good release of ibuprofen in all batches ranging from 49—90.1% after 12 h. In formulation A, batch A1 which was loaded with 1% ibuprofen, gave the highest release of 87.8% at 12 h, while batch A3 containing 2% of ibuprofen gave the least release of 50% at 12 h. Similarly, batch B1 loaded with 1% Ibuprofen, gave the highest release of 98.97% at 12 h while batch B3 containing 2% of ibuprofen gave the least release of 49% at 12 h. However, the formulation with the lipid matrix of *Irvingia wombolu* fat and Phospholipon® 90G (batch B) showed a relatively higher drug release than the formulation with *Irvingia wombolu* fat and moringa oil (batch A). The release of the commercial ibuprofen (Brufen®) used as the reference gave its highest release of 98.87% at 12 h.

### Anti-inflammatory properties of lipid-microspheres

**Fig. 7:**
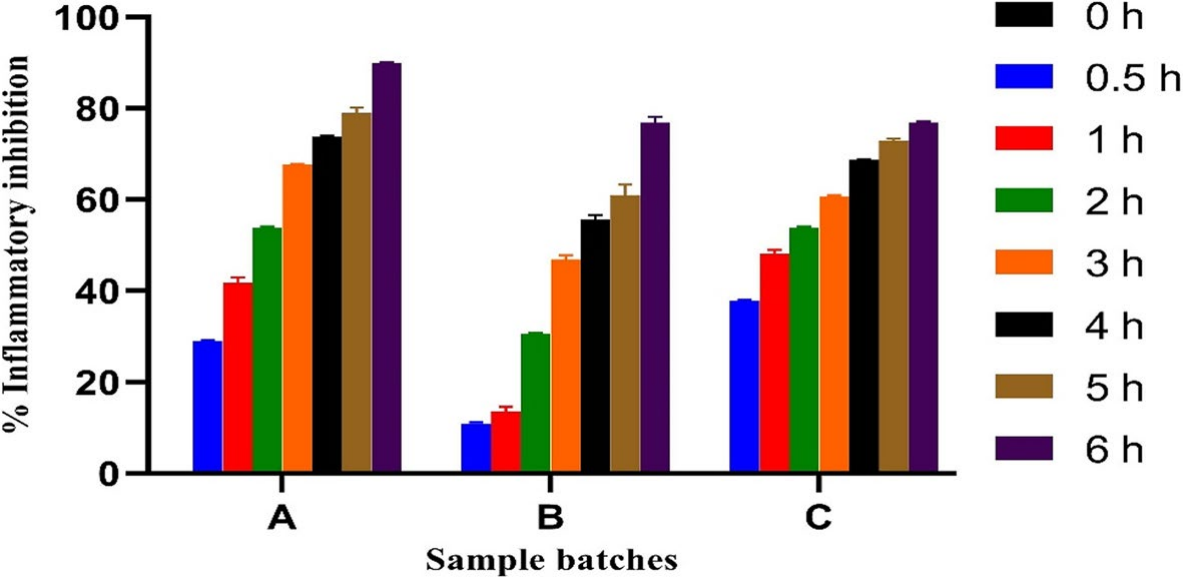
Anti-inflammatory properties if ibuprofen-loaded lipid-microspheres

The results of percentage inflammatory inhibition of ibuprofen-loaded microspheres in Fig. 7 showed that at 0.5 h, the lipid-microspheres least inhibited the size of the oedematous inflammation with 10.97 and 29.10%. Batch A showed good percentage inhibition when compared with the positive control (ibuprofen pure sample) of 37.89% oedema inhibition within the same 0.5 h. The Batch B1 formulated with *Irvingia wombolu* fat and Phospholipon® 90G gave 76.90% oedema inhibition at 6 h while batch A1 formulated with *Irvingia wombolu* fat and moringa oil 89.90% oedema inhibition at 6 h, comparable with the reference drug which had 76.90% oedema inhibition at 6 h. This may be due to an improved oral bioavailability through an enhanced lipids solubility in agreement with an earlier report [36].

### Ulcerogenic properties of the lipid microspheres

The result of percentage ulcer inhibition, showed that the formulation exhibited good gastro-protective properties in addition to its sustained release properties, the ulcer inhibition in Table 6 showed 85 and 72% respectively for the batch B1 and A1 when compared with the positive and negative control.

**Table 6:** Results of ulcerogenic properties of ibuprofen-loaded microspheres

Group	Ulcer score	Ulcer inhibition (%)	Ulcer diameter (mm)
A1	1.03 ± 0.8	72	Lesion < 1
B1	0.99 ± 1.5	85	Lesion < 1
C (reference)	5.04 ± 1.0	10	Lesion ≥ 2
D (control)	0.00 ± 0.00	100	No lesion

Key: A1 and B1 are 1% ibuprofen-loaded lipid-microspheres, C is pure sample of ibuprofen, D is control (normal saline) (*n* = 3 ± SD)

## Discussions

**Fig. 8:**
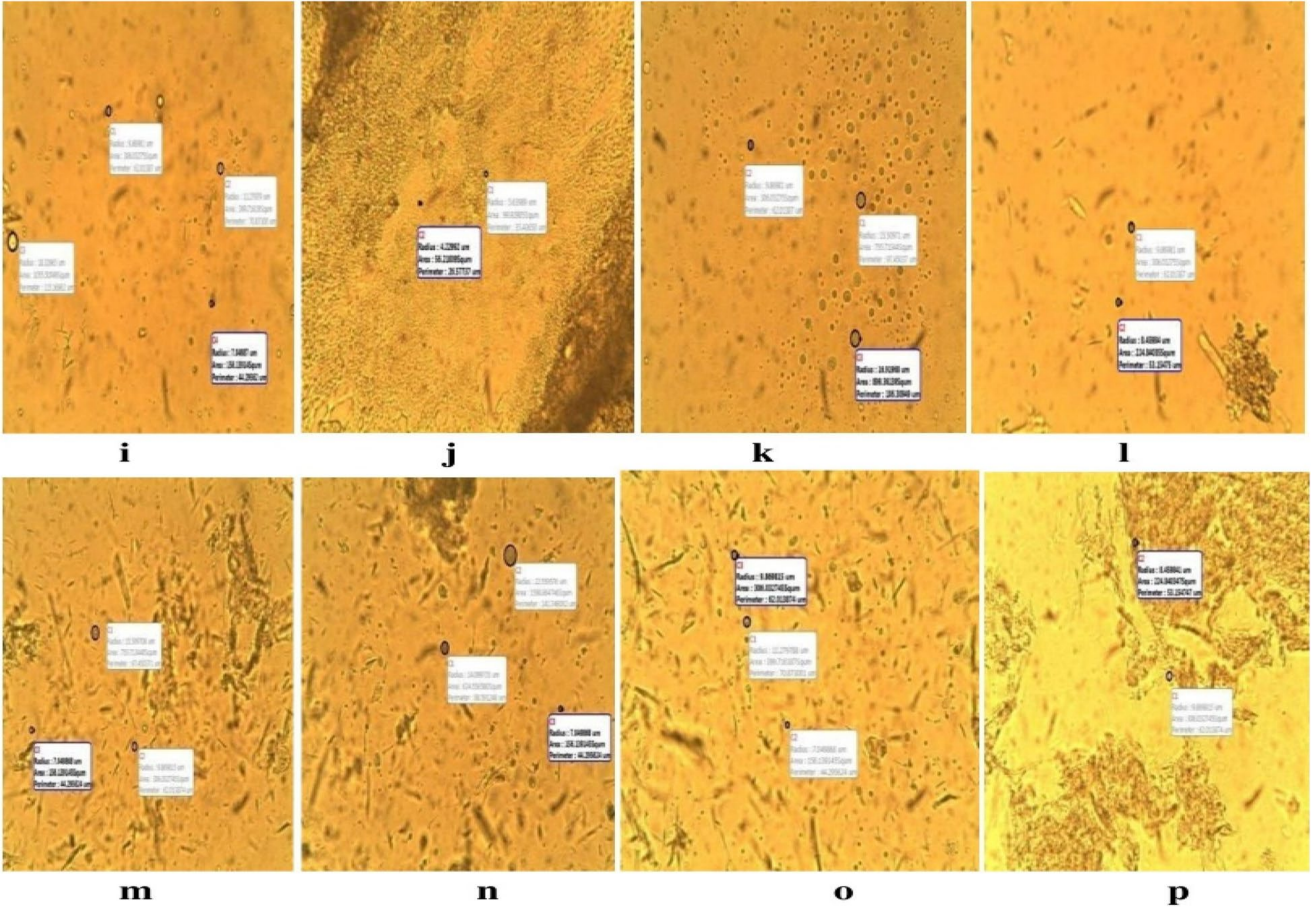
Photomicrographs of lipid-microspheres after lyophilization: (i) 1% ibuprofen lipid microspheres with IRW and MO, (j) 1.5% ibuprofen lipid-microspheres with IRW and MO, (k) 2% ibuprofen with IRW and MO, (l) bland lipid-microspheres with IRW and MO, (m) 1% ibuprofen lipid-microspheres with IRW and PL90G, (n) 1.5% ibuprofen lipid-microspheres with IRW and PL90G, (o) 2% ibuprofen lipid-microspheres with IRW and PL90G, (p) bland ibuprofen lipid-microspheres with IRW and PL90G, IRW = *Irvingia wombolu* fat, MO = moringa oil, PL90G = Phospholipon® 90G

The increased values of the percentage recovery of the lipid microspheres formulated indicates that the formulation technique adopted was reliable and can be reproducible. The change in pH of the unlyophilized lipid microspheres may be due to hydrolytic degradation of some excipients used, hence, suggesting the need to lyophilize the formulation and the possible inclusion of a buffer to maintain the pH. The use of natural oils and fats has been reported in some nanostructured lipid carrier (NLC) formulations majorly in transdermal applications [37]. Lipid drug conjugates are generally used to carry lyophobic drug molecules [38]. These insoluble drug-lipid conjugates had reported better delivery when prepared by its salt formation or through homogenized covalent linkage of the drug and lipids [39]. This suggests less crystallinity and possible retention of an entrapped drug over time. The DSC of the different lipids *Irvingia wombolu* fat, moringa oil and Phospholipon® 90G show endothermic peaks at different temperatures thus signifying the thermal behavior of each excipient used in the formulation process. The thermograms of ibuprofen-loaded lipid-microspheres containing *Irvingia wombolu* fat, moringa oil and phospholipon® 90G in Figs. 5 and 8, show sharp endothermic peaks of ibuprofen corresponding to its crystalinity and melting point as observed in lipid matrices [40]. The varied fatty acids contents of these lipids may have interacted in such a manner as to partly disorder the crystal arrangement of individual lipids. This means that the lipid matrices as shown in Figs. 5 and 8 generated imperfect matrices due to distortion of crystal arrangement of individual lipids after melting and solidification, which may have created numerous spaces for drug localization. However, a minor shift in transition temperature as observed would occur in addition to change in enthalpy of the transition based on the thermotropic behaviour of lipid mixtures [41]. Furthermore, the sharp endothermic peak of Ibuprofen attests to the crystallinity and purity of the drug.

The particle size and distribution could be due to active drug entrapment and encapsulation property. Combination of non-biodegradable natural lipid matrix has shown advantages in drug delivery systems of poorly soluble drugs leading to its bioavailability, prolonged shelf-life, stability and non-toxic properties [22]. The lipid-microspheres formulated with the highest concentration of ibuprofen (2%) in batch A3 and B3 showed the highest mean particle size, this suggests a concentration dependent increase in particle size and reflects the influence of drug concentration on the size of microspheres. The pH and ionic charge of the drugs affect the particle size distribution and kinetics of the lipospheres by the drug ratio to lipid matrix in the formulation [42, 43]. The different particle sizes of lyophilized lipid-microspheres formulated with the lipid matrix *Irvingia wombolu* fat and Phospholipon® 90G (batch B) had a lower particle size than batch A which was formulated with *Irvingia wombolu* fat and moringa oil. These were also at variance within the unlyophilized batch formulations. Therefore, this particle diameter and distribution may result from the following factors; excipients, degree of homogenization, internal force, rate of particle size growth, crystalline habit of the particle size. The mean particle diameter of lipid-microspheres affects the bioavailability of formulated drug and also determines the site of administration of drug formulations. The small particle size of lipid-microspheres (< 20 µm) can be well tolerated by single cell contact, large particle sizes (> 50 µm) are much more reactive due to attractive forces [44].

The increased entrapment efficiency, could be due to an increased surface area of the drug in the medium against saturation effect as reported in drug solubility properties [45], and consequently help to reduce the drug loading concentration with maximum effect and mask toxicity [25]. The batches loaded with 2% ibuprofen (batches A3 and B3) and 1.5% ibuprofen (batches A2 and B2) had the least EE%. This may have arisen from saturation solubility of the lipids thus preventing further entrapment of higher drug concentrations (beyond 1%). However, LC increased with increase in drug loading. The ability of the lipid-microspheres to accommodate active molecules is an important property which could be expressed by the entrapment efficiency and loading capacity.

The in vitro release profile of ibuprofen formulated lipid-microspheres followed first order kinetics. The release of the drug for up to 12 h ensured the possibility of a sustained effect for a longer period and ruled out the possibility of immediate release and dose dumping. Previous studies have shown that there was a burst release from ibuprofen microspheres when evaluated in vitro [46, 47]. This, was attributed to the possibility of the presence of the unloaded drug on the surface of the microspheres. Our formulation, therefore, was able to overcome this issue. In line with earlier reports, ibuprofen lipid-based formulations have shown good sustained release property which could increase its therapeutic effect [48, 49]. The batches of ibuprofen-loaded microspheres formulated with natural oil and fat, showed good anti-inflammatory properties with up to 100% inflammatory inhibition. The inflammatory inhibitions are a confirmation of the ability of ibuprofen as an NSAID to suppress the cascade of physiological and immunological processes caused by mediators and cytokines which can lead to inflammation [50, 51]. The ulcerogenic properties and inhibition potentials of the ibuprofen-loaded microspheres as shown in Table 6, indicate the advantage of these natural lipid matrices for oral drug administration. The batch (B1) formulated with the lipid matrix consisting of *Irvingia wombolu* fat and Phospholipon® 90G showed higher ulcer-inhibition than the batch formulated with *Irvingia wombolu* fat and moringa oil. However, it was observed that the formulations, when administered, had less ulcerogenic effect in comparison with the pure sample of ibuprofen and the negative control. This indicated that formulation of the microparticle reduces the ulcerogenicity of ibuprofen thus providing a better and protective carrier for its delivery. Previous works support this finding [52].

## Conclusion

Ibuprofen-loaded lipid-microspheres were formulated with lipid matrices consisting of *Irvingia wombolu* fat with moringa oil and *Irvingia wombolu* fat with Phospholipon® 90G in the ratio of 2:1 respectively to enhance solubility and bioavailability. In vitro studies carried out showed that ibuprofen-loaded lipid-microspheres exhibited good physicochemical properties and a sustained release property for once daily administration. The formulated lipid-microspheres exhibited good anti-inflammatory properties when compared with the commercial dosage form of ibuprofen in addition to inhibition of the ulcerative effect of ibuprofen. Ibuprofen-loaded lipid-microspheres have advantages over the commercial formulations of ibuprofen which include: low cost of ingredients, low cost of technologies involved in the production (equipment and labour requirement for the production of lipid dosage forms are minimal), little or no ulcerogenicity and better control of inflammation due to an enhanced absorption of drugs from the lipids. The delivery performance of these lipids makes them a better and viable option for the oral delivery of ibuprofen to reduce dosing frequency, enhance its bioavailability and therapeutic effect.

## Data Availability

We declare that all data generated in the course of this research have been properly presented in the manuscript.
